# Prophylactic and long-lasting efficacy of senolytic CAR T cells against age-related metabolic dysfunction

**DOI:** 10.21203/rs.3.rs-3385749/v1

**Published:** 2023-09-26

**Authors:** Corina Amor, Inés Fernández-Maestre, Saria Chowdhury, Yu-Jui Ho, Sandeep Nadella, Courtenay Graham, Sebastian E. Carrasco, Emmanuella Nnuji-John, Judith Feucht, Clemens Hinterleitner, Valentin J.A. Barthet, Jacob A. Boyer, Riccardo Mezzadra, Matthew G Wereski, David A. Tuveson, Ross L. Levine, Lee W Jones, Michel Sadelain, Scott W Lowe

**Affiliations:** 1 Cold Spring Harbor Laboratory. Cold Spring Harbor, NY, USA.; 2 Human Oncology and Pathogenesis Program, Memorial Sloan Kettering Cancer Center, New York, NY, USA.; 3 Louis V. Gerstner Jr Graduate School of Biomedical Sciences, Memorial Sloan Kettering Cancer Center, New York, NY, USA.; 4 Department of Cancer Biology and Genetics. Memorial Sloan Kettering Cancer Center, New York, NY, USA.; 5 Department of Medicine, Memorial Sloan Kettering Cancer Center, New York, NY, USA.; 6 Laboratory of Comparative Pathology. Weill Cornell Medicine, Memorial Sloan Kettering Cancer Center, and Rockefeller University, New York, NY, USA.; 7 Cold Spring Harbor School of Biological Sciences, Cold Spring Harbor, NY, USA.; 8 Center for Cell Engineering, Memorial Sloan Kettering Cancer Center, New York, NY, USA.; 9 Cluster of Excellence iFIT, University Children’s Hospital Tuebingen, Tuebingen, Germany.; 10 Lewis Sigler Institute for Integrative Genomics and Department of Chemistry, Princeton University, Princeton, NJ, USA.; 11 Department of Medicine, Weill Cornell Medical College, New York, NY, USA.; 12 Howard Hughes Medical Institute, Memorial Sloan Kettering Cancer Center, USA.

## Abstract

Senescent cells accumulate in organisms over time because of tissue damage and impaired immune surveillance and contribute to age-related tissue decline^1,2^. In agreement, genetic ablation studies reveal that elimination of senescent cells from aged tissues can ameliorate various age-related pathologies, including metabolic dysfunction and decreased physical fitness^3–7^. While small-molecule drugs capable of eliminating senescent cells (known as ‘senolytics’) partially replicate these phenotypes, many have undefined mechanisms of action and all require continuous administration to be effective. As an alternative approach, we have developed a cell-based senolytic therapy based on chimeric antigen receptor (CAR) T cells targeting uPAR, a cell-surface protein upregulated on senescent cells, and previously showed these can safely and efficiently eliminate senescent cells in young animals and reverse liver fibrosis^8^. We now show that uPAR-positive senescent cells accumulate during physiological aging and that they can be safely targeted with senolytic CAR T cells. Treatment with anti uPAR CAR T cells ameliorates metabolic dysfunction by improving glucose tolerance and exercise capacity in physiological aging as well as in a model of metabolic syndrome. Importantly, a single administration of a low dose of these senolytic CAR T cells is sufficient to achieve long-term therapeutic and preventive effects.

Cellular senescence is a stress response program characterized by stable cell cycle arrest^9,10^ and the production of the senescence-associated secretory phenotype (SASP), which includes proinflammatory cytokines and matrix remodeling enzymes^11^. In physiological conditions in young individuals (e.g., wound healing, tumor suppression), the SASP contributes to the recruitment of immune cells, whose role is to clear the senescent cells and facilitate restoration of tissue homeostasis^11^. However, during aging, the combination of increased tissue damage and decreased function of the immune system leads to the accumulation of senescent cells^1,2^, thereby generating a chronic pro-inflammatory milieu that leads to a range age-related tissue pathologies^5,12–14^. As such, senolytic strategies to eliminate senescent cells from aged tissues have the potential to dramatically improve healthspan.

Most efforts to develop senolytic approaches have focused on the development of small molecule therapies that target as yet poorly defined molecular dependencies present in senescent cells and that must be administered repeatedly over time^15^. In contrast, chimeric antigen receptor T cells (CAR T cells) are a form of cellular therapy that redirects T cell specificity towards cells expressing a specific cell-surface antigen^16^. Unlike small molecules, CAR T cells only require that the target antigen is differentially expressed on target cells compared to normal tissues; moreover, as “living drugs”, these therapeutics have the potential to persist and mediate their potent effects for years after single administration^17^. Leveraging ‘senolytic’ CAR T cells that we previously evaluated in young animals, we set to explore whether CAR T cells could safely and effectively eliminate senescent cells in aged mice and modulate healthspan.

## Results

### uPAR is upregulated in physiological aging

The urokinase plasminogen activator receptor (uPAR) promotes remodeling of the extracellular matrix during fibrinolysis, wound healing and tumorigenesis^18^. In physiological conditions it is primarily expressed in certain subsets of myeloid cells and, at low levels, in the bronchial epithelium^8^. We recently described the upregulation of uPAR on senescent cells across different cell types and multiple triggers of senescence^8^ and showed that CAR T cells targeting this cell-surface protein could efficiently remove senescent cells from tissues in young mice without deleterious effects to normal tissues^8^. Given these results, we wondered whether uPAR might serve as a target for senolytic CAR T cells in aged tissues.

**Extended Data Figure 1 |:**
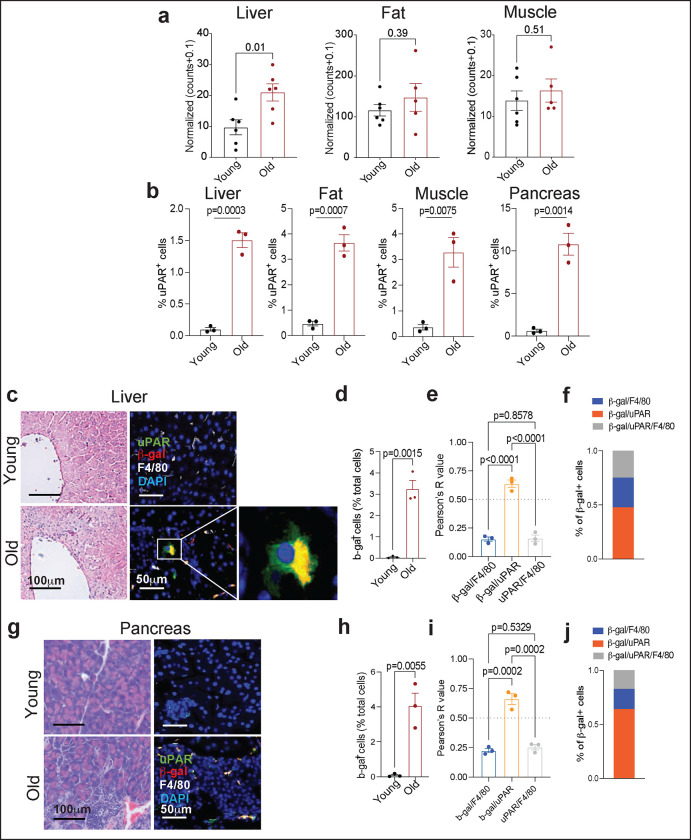
Characterization of uPAR-positive cells in aging. **a,** RNA expression of *Plaur* in liver, adipose tissue (fat) and muscle of young (3 months) or old (21 months) mice. Data obtained from the Tabula Muris Senis project.^22^ **b**, Quantification of immunohistochemical staining of mouse uPAR in liver, adipose tissue, muscle and pancreas from young (age 3 months) or old (age 20 months) mice (n=3 per age). **c**, Hematoxylin and eosin staining and immunofluorescence staining of young (age 3 months n=3 mice) or old (age 18–20 months n=3 mice) livers. uPAR (green), β-gal (red), F4/80 (white), DAPI (blue). **d)** Percentage of SA-b-gal positive cells in young and aged livers in c. **e)** Correlation (Pearson’s R value) of β-gal and F4/80 co-staining, β-gal and uPAR co-staining or uPAR and F4/80 co-staining in aged livers. **f)** Percentage of β-gal positive cells that costain for F4/80, uPAR or uPAR and F4/80 in aged livers. **g)** Hematoxylin and eosin staining and immunofluorescence staining of young (age 3 months n=3 mice) or old (age 18–20 months n=3 mice) pancreas. uPAR (green), β-gal (red), F4/80 (white), DAPI (blue). h) Percentage of SA-b-gal positive cells in young and aged livers in g. i) Correlation (Pearson’s R value) of β-gal and F4/80 co-staining, β-gal and uPAR co-staining or uPAR and F4/80 co-staining in aged pancreas. j) Percentage of β-gal positive cells that costain for F4/80, uPAR or uPAR and F4/80 in aged pancreas. Data are mean ± s.e.m **(a,b,d,e,h,i)**; values are derived from two-tailed unpaired Student’s t-tests **(a,b,d,h)** one-way ANOVA with multiple comparisons **(e,i).** Results are from 1 independent experiment **(a-j).**

**Figure 1 |:**
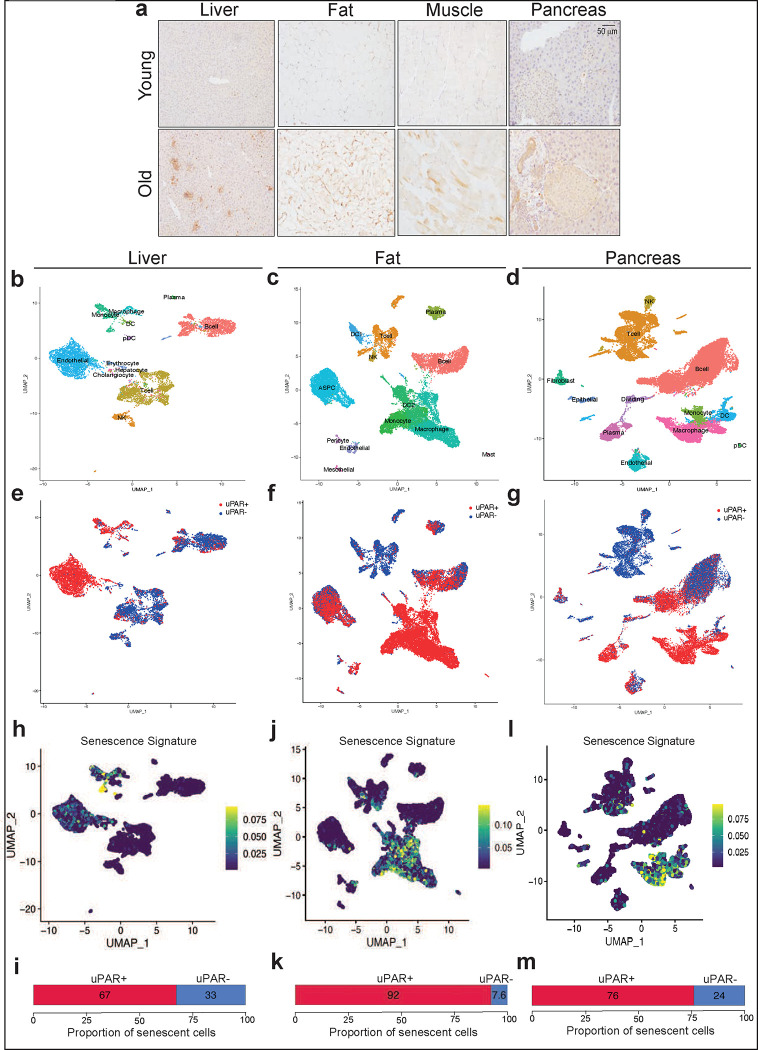
uPAR is upregulated on senescent cells in physiological aging. **a,** Immunohistochemical staining of mouse uPAR in liver, adipose tissue, muscle and pancreas from young (age 3 months) or old (age 20 months) mice (n=3 per age). **b-m,** Single-cell analysis of uPAR expression and senescence. uPAR-positive and uPAR-negative cells were sorted from the liver, adipose tissue and pancreas of 20-month-old mice and subjected to single-cell RNAsequencing by 10X chromium protocol (n=4 mice). **b,** Uniform manifold approximation and projection (UMAP) visualization of liver cell types. **c,** UMAP visualization of adipose tissue cell types. **d,** UMAP visualization of pancreas cell types. **e,** UMAP visualization of hepatic uPAR negative and uPAR positive cell types. **f,** UMAP visualization of adipose uPAR negative and uPAR positive cell types. **g,** UMAP visualization of pancreatic uPAR negative and uPAR positive cell types.**h-m,** UMAP visualizations with senescence signature scores^28^ in each cell indicated by the color scale. Below: quantification of the proportion of uPAR positive and negative cells contributing to the respective senescence signature. **h,i,** liver; **j,k,** adipose tissue; **l,m;** pancreas. Results are from 1 independent experiment **(a-m)**.

Plasma levels of soluble uPAR (suPAR) positively correlate with the pace of aging in humans^19,20^ and *Plaur* (the gene encoding uPAR) is a component of the SenMayo gene signature recently reported to identify senescent cells in aging^21^. To explore the association with uPAR expression in aged tissues further, we surveyed RNA-sequencing (RNA-seq) data from the Tabula Muris Senis project^22^. Expression of *Plaur* was upregulated in several organs (e.g.: liver, adipose tissue) in samples from 20-month-old mice compared to 3-month-old mice (Extended Data Fig. 1a). Because mRNA levels are not linearly related to surface protein levels^23^, we performed immunohistochemistry and indeed confirmed an age-associated increase in uPAR protein in liver, adipose tissue, skeletal muscle and pancreas (Fig. 1a and Extended Data Fig. 1b). This increase in fraction of uPAR-positive cells was paralleled by an increase in the percentage of SA-β-gal positive cells (Extended Data Fig.1c,d,g,h). Co-immunofluorescence revealed that the vast majority of these SA-β-gal expressing cells were in fact uPAR positive (Extended Data Fig.1e,i). Only a minority of these cells were macrophages as evidenced by co-expression of F4/80, though it was notable that those macrophages that were β-gal/uPAR double-positive were extraordinarily rare in young mice and also markedly increased with age (Extended Data Fig.1e,f,i,j).

**Extended Data Figure 2 |:**
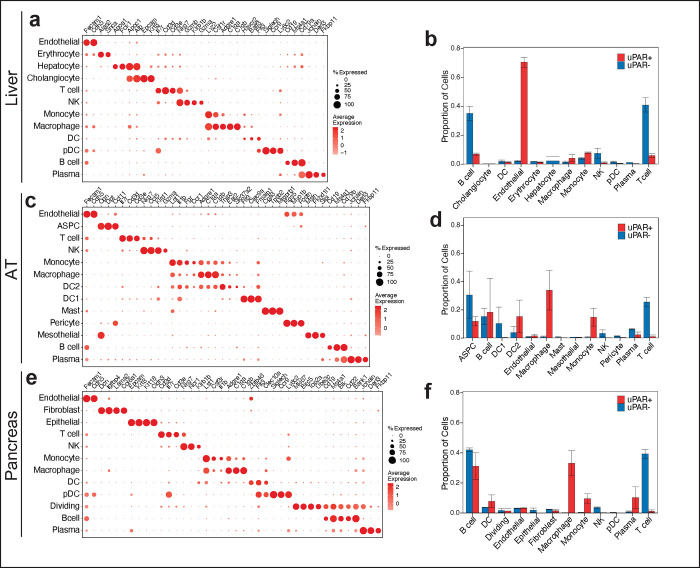
Single cell profile of aged tissues. **a**, Dot plot showing expression of 34 signature genes across the 12 lineages of the liver. The size of the dots represents the proportion of cells expressing a particular marker, and the color scale indicates the mean expression levels of the markers (z-score transformed). **b**, Fractions of uPAR-positive and uPAR-negative cells in the various lineages in liver (n=4 mice per group). Error bars represent s.d. c, Dot plot showing expression of 40 signature gene expressions across the 13 lineages of the adipose tissue. The size of the dots represents the proportion of cells expressing a particular marker, and the color scale indicates the mean expression levels of the markers (z-score transformed). **d**, Fractions of uPAR-positive and uPAR-negative cells in the various lineages in adipose tissue (n=4 mice per group). Error bars represent s.d. **e**, Dot plot showing expression of 40 genes across the 12 lineages of the pancreas. The size of the dots represents the proportion of cells expressing a particular marker, and the color scale indicates the mean expression levels of the markers (z-score transformed). **f**, Fractions of uPAR-positive and uPAR-negative cells in the various lineages in pancreas (n=4 mice per group). Error bars represent s.d. Data are mean ± s.d.; p values are derived from two-tailed unpaired Student’s t-tests **(b,d,f).** Results are from 1 independent experiment **(a-f).**

**Extended Data Figure 3 |:**
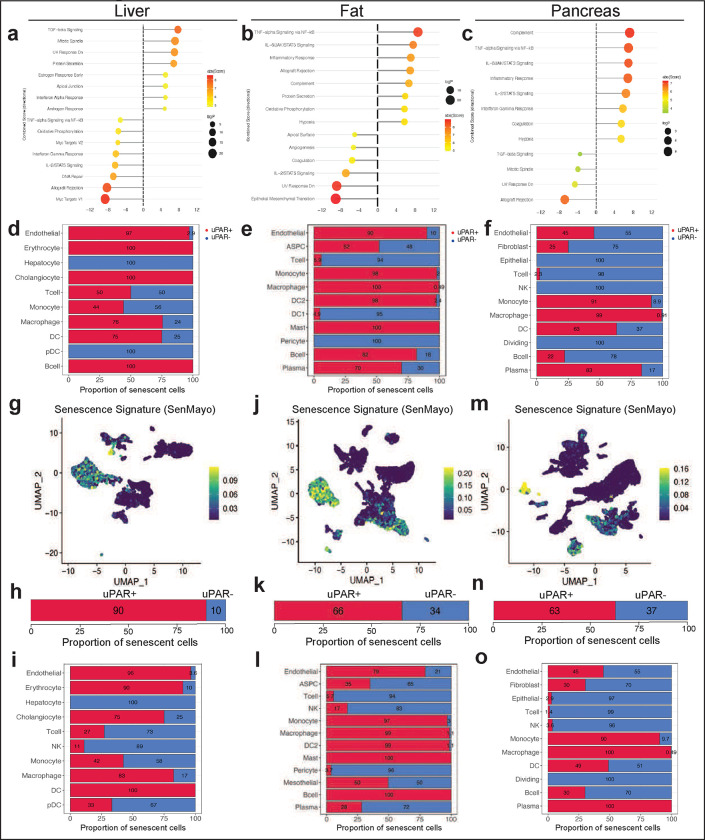
Characteristics of senescent uPAR-positive cells in aged tissues. **a-c,** Molecular Signature Database Hallmark 2020 signatures that are significantly enriched in uPAR positive cells vs uPAR negative cells of liver **(a),** adipose tissue **(b)** and pancreas **(c). d-f,** quantification of the proportion of uPAR positive and negative cells by cell type contributing to the respective senescence signature in Fig.1h **(d)**, Fig.1j **(e)** and Fig.1l **(f)**. **g-o,** UMAP visualizations with senescence signature scores^21^ in each cell indicated by the color scale. Below: quantification of the proportion of uPAR positive and negative cells contributing to the respective senescence signature in total **(h,k,n)** and by cell type **(I,l,o). g,h,I,** liver; **j,k,l,** adipose tissue; **m,n,o;** pancreas. Results are from 1 independent experiment **(a-m)**.

To add granularity to our understanding of the molecular characteristics of uPAR-positive cells in aged tissues, we performed single-cell RNA sequencing (scRNAseq) on approximately 4,000–15,000 uPAR-positive and -negative cells FACS sorted from the liver, fat and pancreas (Fig. 1b–m and Extended Data Fig. 2 and 3). Using unsupervised clustering and marker-based cell labelling^24,25^, we identified distinct cell types and states in each tissue (Fig. 1b–d and Extended Data Fig.2). Thus, our isolation strategy and sequencing identified the major cell types present in each of the three organs. Of note, some minor cell types (e.g., hepatic stellate cells in the liver, and beta cells in the pancreas) require specialized isolation procedures and were not captured using our protocol ^26,27^.

Analysis of the different populations for uPAR expression indicated that endothelial and myeloid cells were the most prominent uPAR-expressing populations in the liver (Fig. 1e and Extended Data Fig. 2b), whereas in adipose tissue uPAR was expressed mainly in subsets of preadipocytes, dendritic cells and myeloid cells (Fig. 1f and Extended Data Fig. 2d). In the aged pancreas, uPAR expression was prominent in subsets of endothelial cells, fibroblasts, dendritic cells and myeloid cells (Fig. 1g and Extended Data Fig. 2f). Compared to to uPAR negative cells, uPAR positive cells, were significantly enriched in gene signatures linked to inflammation, the complement, and the coagulation cascade as well as TGFb signaling (Extended Data Fig.3a–c).

Importantly, when senescent cells present in these tissues were identified using two independent transcriptomic signatures of senescence^21,28^, we observed that the main senescent cells types present in aged livers were indeed endothelial and myeloid cells (Fig. 1h and Extended Data Fig.3d,g–i), in adipose tissue were dendritic cells, myeloid cells and preadipocytes (Fig. 1j and Extended Data Fig.3e,j–l) and in the pancreas were endothelial, fibroblasts, dendritic and myeloid cells (Fig. 1l and Extended Data Fig.3f, m–o). Thus, uPAR positive cells constituted a significant fraction of the senescent-cell burden in these tissues (67–90% in liver, 92–66% in adipose tissue and 76–63% in pancreas) (Fig.1i,k,m and Extended Data Fig. 3h,k,n). Note that while our analysis could not evaluate pancreatic beta cells, analysis of published data revealed that expression of *Plaur* was significantly upregulated in senescent beta cell populations isolated from aged animals and subjected to bulk RNA-seq^7^.

**Extended Data Figure 4|:**
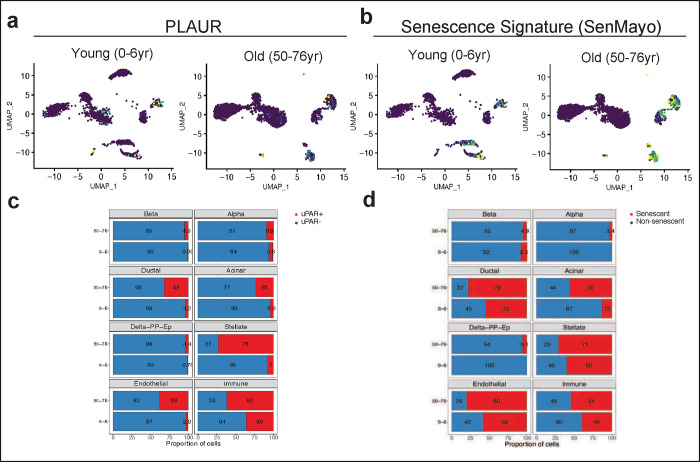
Upregulation of uPAR and senescence signatures in aged human pancreas. Single-cell RNAsequencing data of human pancreas of different ages from^29^ was analyzed. **a,** Uniform manifold approximation and projection (UMAP) visualization of *Plaur* expression across pancreas cell types in young humans (0–6 years old) and old humans (50–76 years old).**b,** UMAP visualization of senescence signature expression^21^ across pancreas cell types in young humans (0–6 years old) and old humans (50–76 years old). **c,** Quantification of the proportion of uPAR positive and negative cells by cell type and age. **d,** Quantification of the proportion of senescent signature expressing or non-expressing cells cells by cell type and age.

Finally, to ascertain whether uPAR was expressed in senescent cells that accumulate with age in human tissues, we analyzed available datasets of human pancreas collected from young (0–6 year old) and aged (50–76 year old) individuals ^29^. While we were limited to an analysis of *Plaur* transcript abundance in these settings, we found that the fraction of Plaur-expressing cells was substantially greater in older individuals (Extended Data Fig.4).

Overall, these results indicate that the levels of uPAR positive senescent cells increase with age and that most senescent cells present in aged tissues express uPAR. The fact that we can identify settings in which an increased expression of uPAR protein expression doesn’t correlate with *Plaur* levels indicates that, in the context of aging and perhaps other settings, the absence of an induction of *Plaur* transcript levels does not exclude the possibility of an increase in uPAR protein expression.

### Effect of uPAR CAR T cells in naturally aged mice

**Figure 2 |:**
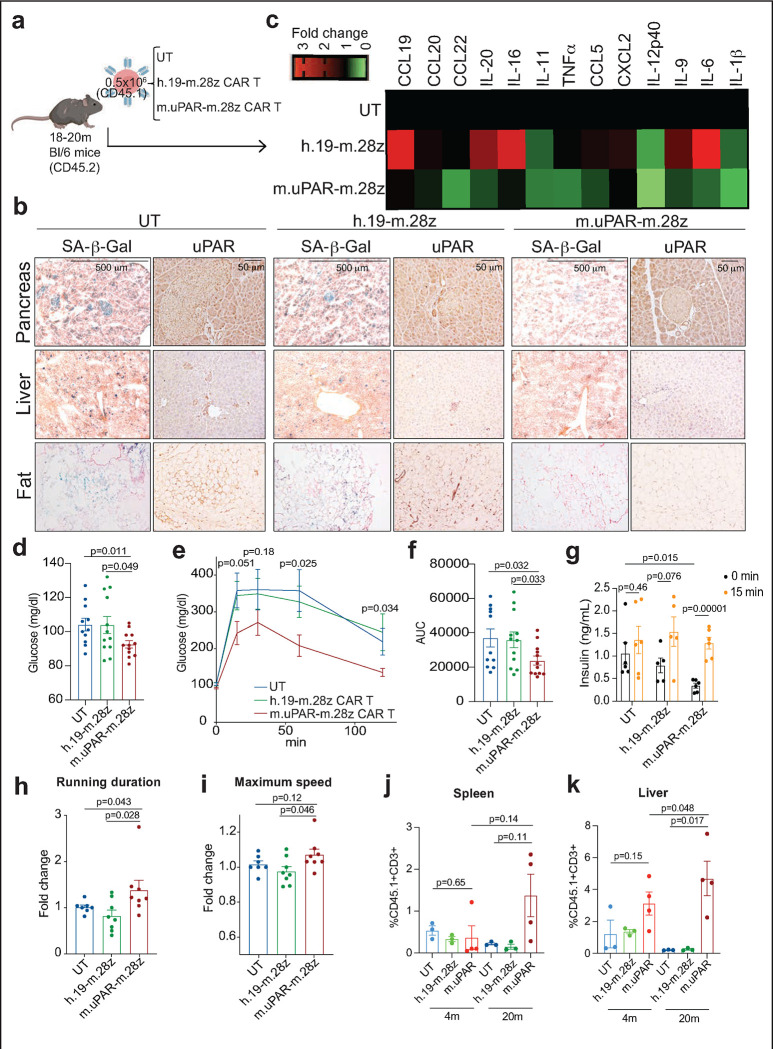
uPAR CAR T cells revert natural age-associated phenotypes. **a,** Experimental scheme for Fig. 2b–k. 18- to 20-month-old C57Bl/6N mice were injected with 0.5×10^6^ m.uPAR-m.28z CAR T cells, h.19-m.28z CAR T cells, or untransduced T (UT) cells generated from CD45.1 mice 16h after administration of cyclophosphamide (200 mg/kg). Mice were monitored over time and/or harvested 20 days after cell infusion. Schematic was created with BioRender.com. **b,** Representative staining of SA-β-Gal and uPAR 20 days after cell infusion. **c,** Heatmap depicting fold change in the levels of SASP cytokines compared to UT treated mice (n=3 for untransduced T cells; n=3 for h.19-m.28z; n=4 for m.uPAR-m.28z). **d,** Levels of basal glucose (mg/ml) after starvation 2.5 months after cell infusion (n=11 mice for untransduced T cells; n=12 for h.19-m.28z and for m.uPAR-m.28z). **e,** Levels of glucose before (0 min) and after intraperitoneal administration of glucose (2 g/kg) 2.5 months after cell infusion (samples sizes as in **d**). **f,** Area under the curve (AUC) representing the results from **e**. Each point represents a single mouse. **g,** Levels of insulin before and 15 minutes after intraperitoneal glucose administration (2 g/kg) 2.5 months after cell infusion (n=6 for untransduced T cells; n=5 for h.19-m.28z; n=6 for m.uPAR-m.28z). **h,** Fold change in time to exhaustion in exercise capacity testing before cell infusion and 2.5 months after it (n=7 for untransduced T cells; n=8 for h.19-m.28z and n= 8 for m.uPAR-m.28z). **i,** Fold change in maximum speed in capacity testing before cell infusion and 2.5 months after it (sample sizes as in **h**). **j,k,** Percentage of CD45.1^+^ T cells in the spleen (**j**) or liver (**k**) of 4-month-old or 20-month-old mice 20 days after cell infusion (n=3 mice per age group for untransduced T cells and for h.19-m.28z; n=4 for m.uPAR-m.28z). Results are from 2 independent experiments **(d-f; h-i)** or 1 experiment **(b-c; g; j-k).** Data are mean ± s.e.m.; p values from two-tailed unpaired Student’s t-test **(d-g;j-k)** or. Mann Whitney test **(h,i).**

To determine the tolerability and therapeutic activity of uPAR-targeting CAR T cells on physiologically aged mice, we intravenously infused aged C57BL/6 mice (18–20 months old) with our previously developed murine second-generation CAR T cells targeting mouse uPAR^8^ (m.uPAR-m.28z). m.uPAR-m.28z CAR T cells contain an anti-mouse uPAR single-chain variable fragment (scFV) linked to mouse CD28 costimulatory and mouse CD3ζ signaling domains and are therefore fully murine CAR T cells that allow for syngeneic studies^8^. Importantly, the CAR T cells were generated from CD45.1 mice and infused into C57BL/6 mice which are CD45.2, thus allowing for CAR T cells to be differentiated from endogenous T cells and therefore monitored over time (Fig. 2a). As controls, parallel cohorts of sex and aged matched mice were infused with the same dose of either untransduced T (UT) cells or T cells expressing a murine CAR targeting human CD19 (h.19-m.28z) that does not recognize the murine CD19 protein but encompasses the exact same signaling structure thus controlling for non-specific T cell cytotoxicity. We opted to test a dose of 0.5×10^6^ CAR-positive cells, which we previously found to balance safety and senolytic efficacy in young animals^8^.

**Extended Data Figure 5 |:**
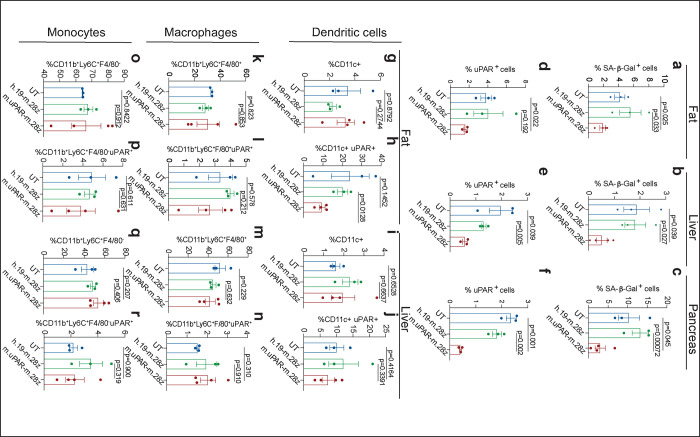
Effect of uPAR CAR T cells on aged tissues. **a-c,** Quantification of SA-β-Gal–positive cells in adipose tissue, liver and pancreas 20 days after cell infusion (n=3 for UT; n=3 for h.19-m.28z; n=4 for m.uPAR-m.28z). **d-f**, Quantification of uPAR-positive cells in adipose tissue, liver and pancreas 20 days after cell infusion (n=3 per group). **g-j**, Percentage of dendritic cells and uPAR^+^ dendritic cells in the adipose tissue **(g,h)** or liver **(i,j)** 20 days after cell infusion (n=3 for UT; n=3 for h.19-m.28z; n=4 for m.uPAR-m.28z). **k-n**, Percentage of macrophages and uPAR^+^ macrophages in the adipose tissue **(k,l,)** or liver (**m,n)** 20 days after cell infusion (n=3 for UT; n=3 for h.19-m.28z; n=4 for m.uPAR-m.28z). **o-r,** Percentage of monocytes and uPAR^+^ monocytes in the adipose tissue **(o,p)** or liver **(q,r)** 20 days after cell infusion (n=3 for UT; n=3 for h.19-m.28z; n=4 for m.uPAR-m.28z). Results of 1 independent experiment **(a-r).** Data are mean ± s.e.m.; p values from two-tailed unpaired Student’s t-test **(a-r)**.

Mice infused with m.uPAR-m.28z CAR T cells, but not controls, showed a reduction in the proportions of SA-β-Gal- and uPAR-positive cells throughout the tissues examined, most notably in the pancreas, liver and adipose tissue (Fig. 2b and Extended Data Fig. 5). As has been previously reported, our aged mouse cohort displayed elevated levels of pro-inflammatory cytokines linked to the SASP in the peripheral blood, a phenomenon often referred to as “inflammaging”^30^. Consistent with a reduction in senescent cell burden and/or improved organismal health, uPAR-m.28z CAR T treated animals showed a significant decrease in the plasma levels of these factors (Fig. 2c).

**Extended Data Figure 6 |:**
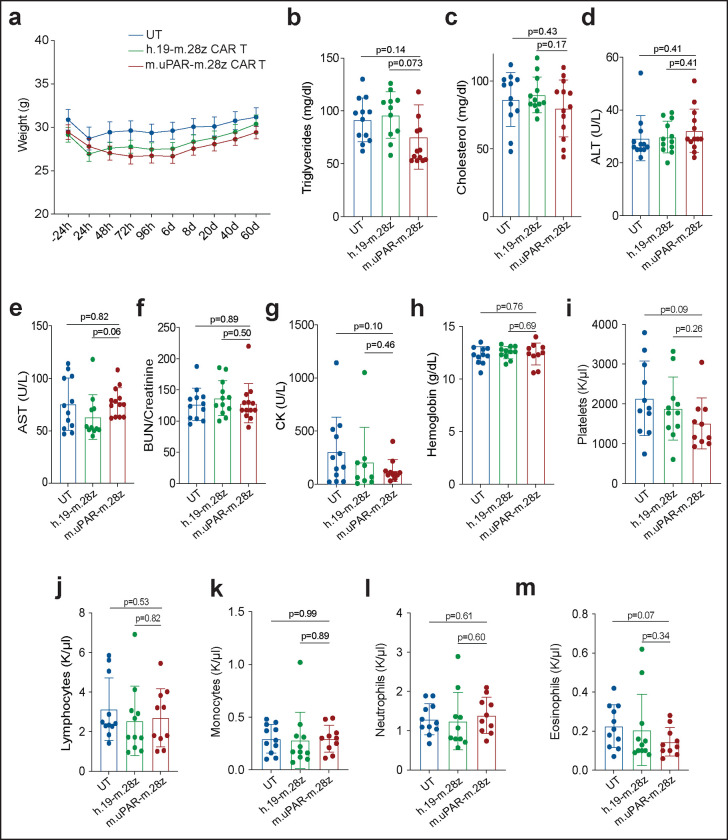
Safety of uPAR CAR T cells in aged mice. Mice received cell infusions at 18–20 months. **a,** Weight of mice 24h before and at different time points after cell infusion (n=12 mice for untransduced T cells [UT]; n=11 for h.19-m.28z; n=12 for m.uPAR-m.28z). **b,** Levels of triglycerides 20 days after cell infusion (n=12 mice for UT; n=11 for h.19-m.28z; n=13 for m.uPAR-m.28z). **c,** Levels of cholesterol 20 days after cell infusion (n=12 for UT and for h.19-m.28z; n=13 for m.uPAR-m.28z). **d,** Levels of ALT 20 days after cell infusion (sample sizes as in **c**). **e,** Levels of AST 20 days after cell infusion (n=12 for UT; n=11 for h.19-m.28z; n=13 for m.uPAR-m.28z). **f,** BUN/creatinine ratio 20 days after cell infusion (sample sizes as in **c**). **g,** Creatine kinase (CK) 20 days after cell infusion (n=12 for UT; n=9 for h.19-m.28z; n=11 for m.uPAR-m.28z). **h,** Levels of hemoglobin 20 days after cell infusion (n=11 for UT; n=11 for h.19-m.28z; n=10 for m.uPAR-m.28z). **i,** Number of platelets 20 days after cell infusion (n=11 for UT; n=11 for h.19-m.28z; n=10 for m.uPAR-m.28z). **j,** Number of lymphocytes 20 days after cell infusion (n=11 for UT; n=11 for h.19-m.28z; n=10 for m.uPAR-m.28z). **k,** Number of monocytes 20 days after cell infusion (n=11 for UT; n=11 for h.19-m.28z; n=10 for m.uPAR-m.28z). **l,** Number of neutrophils 20 days after cell infusion (n=11 for UT; n=10 for h.19-m.28z; n=10 for m.uPAR-m.28z). **m,** Number of eosinophils 20 days after cell infusion (n=11 for UT; n=11 for h.19-m.28z; n=10 for m.uPAR-m.28z). Results for all panels are from 2 independent experiments**.** Data are mean ± s.e.m.; p values from two-tailed unpaired Student’s t-test **(b-m)**.

**Extended Data Figure 7 |:**
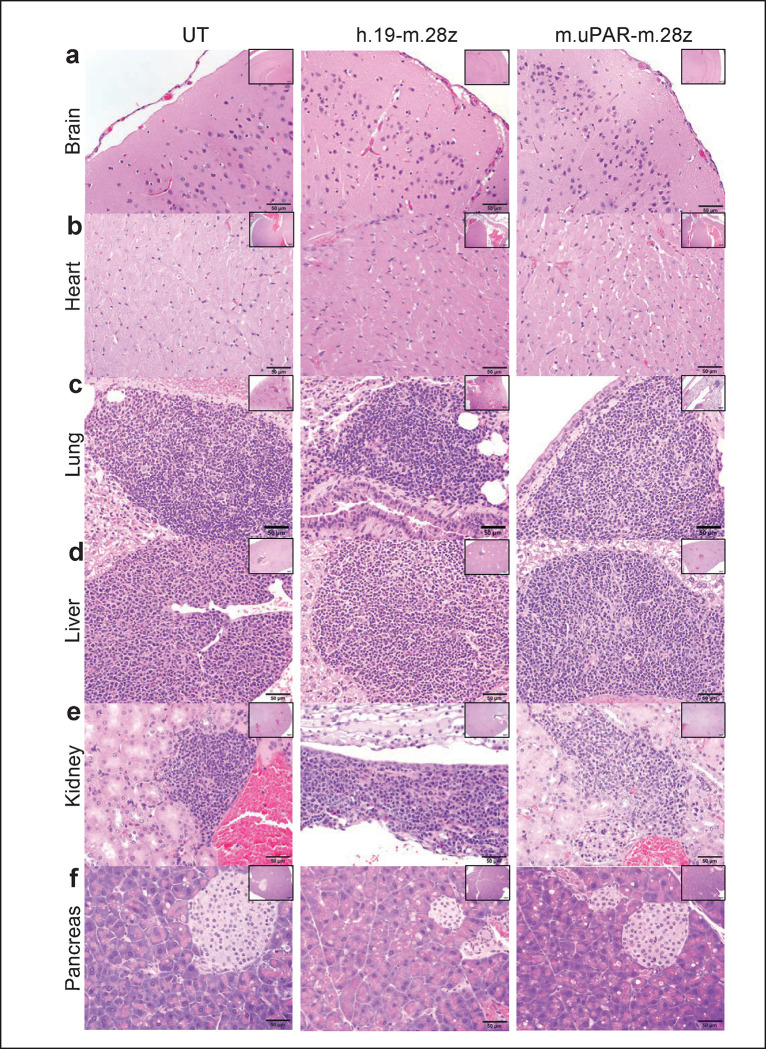
uPAR CAR T cells are not associated with signs of tissue damage in aged tissues and do not exacerbate spontaneous age-related histological changes in lung, liver and kidneys. Mice received cell infusions at 18–20 months and were sacrificed 20 days after infusion of the indicated T cells. Sections were stained with hematoxylin and eosin. Aged mice showed mononuclear leukocytic aggregates composed predominantly of lymphocytes and plasma cells in tissues in an age dependent manner. These leukocytic aggregates were more frequently observed in tissues from uPAR-m.28z CAR T- treated aged mice than tissues from control aged mice and were not associated with necrosis and/or degeneration in tissues from both experimental and control aged mice. These lymphocytic and plasmocytic aggregates in tissues are often observed in naïve aged mice and are considered spontaneous background findings in longitudinal aging studies in mice^50,51^. **a**, Representative sections of normal cerebral cortex and meninges at the level of the posterior hypothalamus (inset: hippocampus). **b**. Histology of normal cardiomyocytes and interstitium in myocardium (inset: ventricles and interventricular septum). **c**. Representative histology of normal lungs showed dense aggregates of lymphocytes and fewer plasma cells and macrophages around bronchioles or vasculature (inset: pulmonary lobes). **d**. The liver from aged mice showed accumulation of lymphocytic and histiocytic aggregates in portal to periportal regions (Inset: hepatic lobe). **e**. Histology of the kidneys showed accumulation of lymphocytes and plasma cells in the renal interstitium (n & o) and around blood vessels (inset: renal cortex, medulla and pelvis). **f**. Representative sections of normal pancreatic acini (exocrine pancreas) and islets of Langerhans (endocrine pancreas; inset: pancreatic lobule). Images were captured at 4x (insets) and 40x magnifications.

Despite detectable expression of uPAR in some normal tissues, our previous work indicates that a dose of 0.5×10^6^ m.uPAR-m.28z CAR T cells is well tolerated in young mice^8^. As was the case in young animals, the dose of 0.5×10^6^ m.uPAR-m.28z CAR T cells was well tolerated in aged mice (18–20 months old), all of whom remained active without observable signs of morbidity, weight loss, or relevant alterations in serum chemistry or complete blood counts (Extended Data Fig. 6). In addition, microscopic evaluation of tissues did not reveal tissue damage secondary to toxicity in aged tissues obtained from whole body necropsies of m.uPAR-m.28z CAR T treated mice when compared to age-matched control treated animals (Extended Data Fig. 7).

**Extended Data Figure 8 |:**
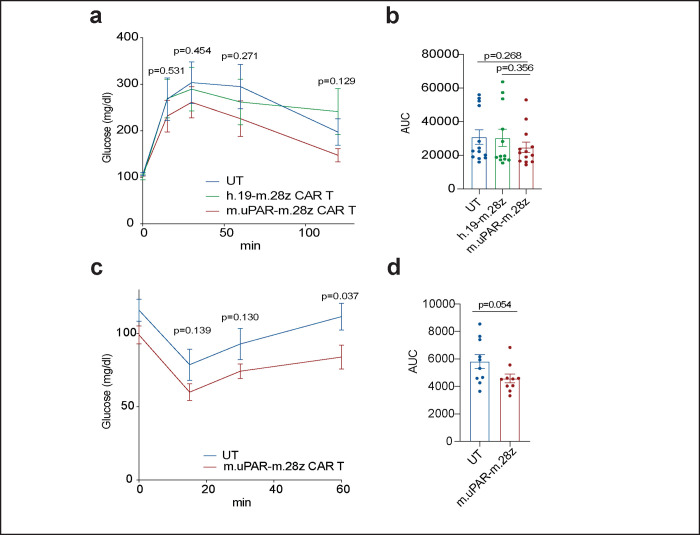
Effect of uPAR CAR T cells in young and old tissues. **a-b,** Mice received cell infusion at 3 months old. **a,** Levels of glucose before (0 min) and after intraperitoneal administration of glucose (2 g/kg) 2.5 months after cell infusion (n=13 for untransduced T cells; n=12 for h.19-m.28z and n=13 for m.uPAR-m.28z). **b,** Area under the curve (AUC) representing the results from **a**. Each point represents a single mouse. **c-d,** Mice received cell infusion at 18–20 months old. **c,** Levels of glucose before (0 min) and after intraperitoneal administration of insulin (0.5 units/kg body weight) 2.5 months after cell infusion (n=10 for untransduced T cells and n=10 for m.uPAR-m.28z). **d,** Area under the curve (AUC) representing the results from **c**. Each point represents a single mouse. Results of 2 independent experiments **(a,b)** or 1 independent experiment **(c,d)**. Data are mean ± s.e.m.; p values from two-tailed unpaired Student’s t-test **(a-d)**.

One prominent feature of aging in humans and mice is the emergence of age-related metabolic dysfunction, which is a collection of phenotypes linked to impaired glucose tolerance^7,31^ and decreased exercise capacity^3,32^. Interestingly, we observed that aged m.uPAR-m.28z CAR T treated mice had significantly decreased fasting glucose levels compared with UT or h.19-m.28-z-treated controls (Fig. 2d). Upon challenge with an intraperitoneal bolus of glucose (2 g/kg), m.uPAR-m.28z CAR T treated aged but not young mice presented significantly lower plasma glucose levels than controls for over 2 hours after administration (Fig. 2e,f and Extended Data Fig. 8a,b). Furthermore, m.uPAR-m.28z CAR T treated mice had lower basal insulin levels after fasting that was followed by a significant increase in insulin levels 15 minutes after the glucose load, indicative of improved pancreatic β cell function (Fig. 2g). Of note, m.uPAR-m.28z CAR T treated aged mice also presented improved peripheral insulin sensitivity, suggesting a coordinated multiorgan improvement in glucose homeostasis (Extended Data Fig. 8c,d). In addition, most aged mice with m.uPAR-m.28z CAR T showed improvements in their exercise capacity at 2.5 months after treatment compared to pretreatment levels (Fig. 2h,i).

**Extended Data Figure 9 |:**
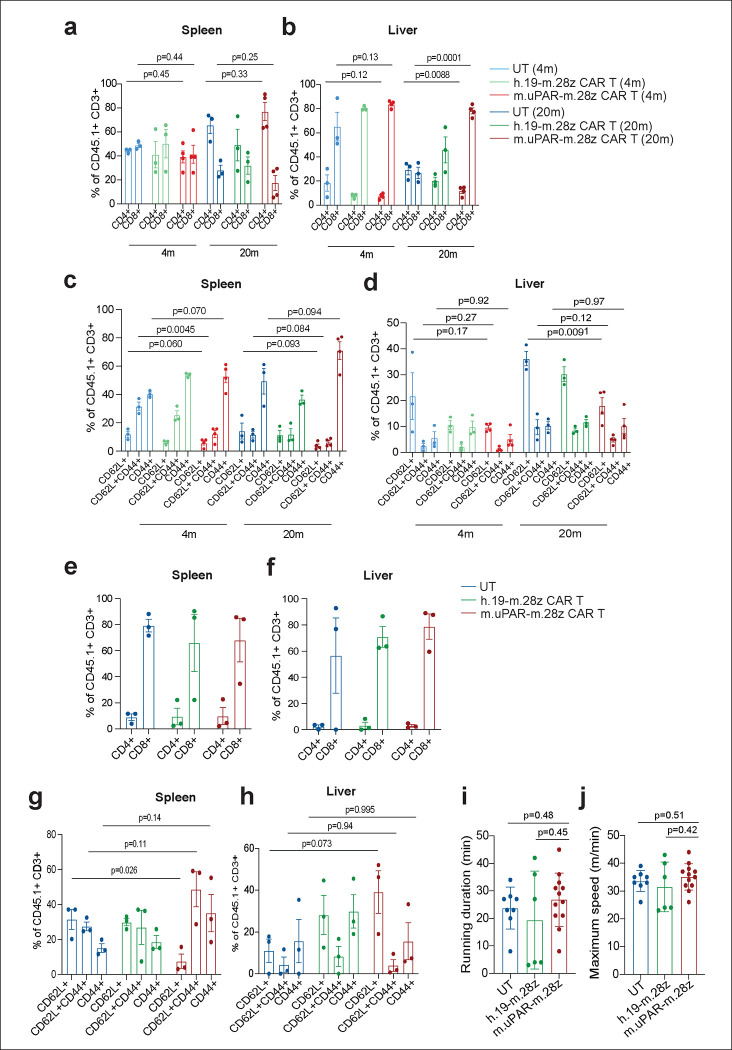
Profile of uPAR CAR T cells in aging. **a,b,** Percentage of CD4^+^ or CD8^+^ cells among CD45.1^+^ T cells from the spleen (**a**) or liver (**b**) of 4-month-old or 20-month-old mice 20 days after cell infusion (n=3 mice per age group for untransduced T cells [UT] and for h.19-m.28z; n=4 for m.uPAR-m.28z). **c,d,** Percentage of CD45.1^+^ T cells expressing differentiation markers CD62L and CD44 in the spleen (**c**) or liver (**d**) of 4-month-old or 20-month-old mice 20 days after cell infusion (sample sizes as in **a**). **e,f,** Percentage of CD4^+^ or CD8^+^ cells among CD45.1^+^ T cells in the spleen (**e**) or liver (**f**) of 15-month-old mice 12 months after cell infusion (n=3 mice per group). **g,h,** Percentage of CD45.1^+^ T cells expressing differentiation markers CD62L and CD44 on CD45.1^+^ T cells in the spleen (**g**) or liver (**h**) of 15-month-old mice 12 months after cell infusion (n=3 mice per group). **i,** Time to exhaustion in exercise capacity testing 12 months after cell infusion (n=8 for untransduced T cells; n=6 for h.19-m.28z; n=12 for m.uPAR-m.28z). **j,** Maximum speed (m/min) in capacity testing 12 months after cell infusion (sample sizes as in **i**). Results of 1 independent experiment **(a-h)** or 2 independent experiments **(i,j)**. Data are mean ± s.e.m.; p values are from two-tailed unpaired Student’s t-test **(a-h)** or Mann Whitney test **(i,j).**

**Figure 3 |:**
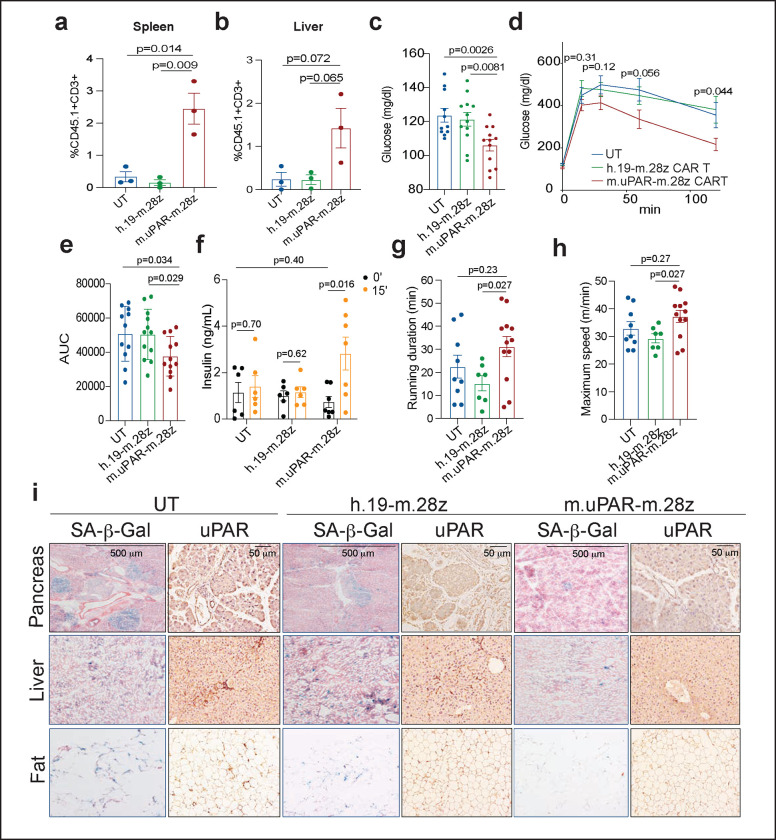
uPAR CAR T cells prevent natural age-associated phenotypes. 3–4-month-old C57Bl/6N mice were injected with 0.5×10^6^ m.uPAR-m.28z CAR T cells, h.19-m.28z CAR T cells, or untransduced T cells generated from CD45.1 mice 16h after administration of cyclophosphamide (200 mg/kg). Mice were monitored over time and/or harvested at 15 months of age. **a,b,** Percentage of CD45.1^+^ T cells in the spleen (**a**) or liver (**b**) of 15-month-old mice 12 months after cell infusion (n=3 mice per group). **c,** Levels of basal glucose after starvation 15–18 months after cell infusion (n=11 mice for untransduced T cells; n=12 for h.19-m.28z and for m.uPAR-m.28z). **d,** Levels of glucose before (0 min) and after intraperitoneal administration of glucose (2 g/kg) 15–18 months after cell infusion (sample sizes as in **c**). **e,** Area under the curve (AUC) representing the results from **d**. Each point represents a single mouse. **f,** Levels of insulin (ng/ml) before and 15 minutes after intraperitoneal glucose (2 g/kg) 15 months after cell infusion (n=6 for untransduced T cells; n=6 for h.19-m.28z; n=7 for m.uPAR-m.28z). **g,** Time to exhaustion in exercise capacity testing 6 months after cell infusion (n=9 for untransduced T cells; n=7 for h.19-m.28z; n=12 for m.uPAR-m.28z). **h,** Maximum speed (m/min) in capacity testing 6 months after cell infusion (sample sizes as in **g**). **i,** Representative staining of SA-β-Gal and uPAR 15 months after cell infusion. Results are from 1 independent experiment **(a-b; f; i)** or 2 independent experiments **(c-e; g-h).** Data are mean ± s.e.m.; p values from two-tailed unpaired Student’s t-test **(a-f)** or Mann Whitney test **(g,h). **

Importantly, the improvement in metabolic function noted in m.uPAR-m.28z CAR T cell-treated old mice was accompanied by a significant expansion of m.uPAR-m.28z CAR T cells and their trafficking to several organs such as liver and spleen as assessed by flow cytometry (Fig. 2j,k). These m.uPAR-m.28z CAR T cells were mostly cytotoxic CD8+ T cells in the livers and CD4+ T cells in the spleen and presented an effector phenotype indicative of their activated response (Extended Data Fig. 9a–d). Of note, this expansion did not occur in aged-matched UT or h.19-m.28z-treated controls and was significantly lower in m.uPAR-m.28z CAR T treated young mice, results that were consistent with the lower fraction of uPAR-positive cells in younger animals (Fig. 1a; Fig. 3a,b and Extended Data Fig. 1).

Collectively, these results show that uPAR CAR T cells can safely and effectively remove senescent uPAR-positive cells in the tissues of naturally aged mice and ameliorate age-dependent metabolic and physical dysfunction.

### Persistence and preventive activity of uPAR CAR T cells during physiological aging.

Unlike small molecules, CAR T cells can persist in the organism and exert their effects over time^17^. Indeed, in human cancer patients cured of disease, the presence of CAR T cells has been noted as much as 10 years after the initial infusion^17^. Such persistence raises the question of whether the administration of uPAR CAR T cells in young animals would prevent or delay the development of age-triggered phenotypes later in life. To explore this possibility, we infused young mice (3 months old) with one dose of 0.5×10^6^ m.uPAR-m.28z CAR T, h.19-m.28z CAR T or UT cells and monitored the mice over their natural lifespan (Fig. 3). Despite the initially lower numbers of uPAR-positive cells compared to aged animals (see above), uPAR CAR T cells were detectable in the spleens and livers of treated mice 12 months after the initial single infusion at significantly higher levels than the low number of persisting UT or h.19 CAR T controls (Fig. 3a,b). Consistent with their persistent activity, flow cytometry of the spleen and livers of uPAR CAR T cell treated mice indicated that the persisting cells were mostly cytotoxic CD8 T cells harboring a memory and effector phenotype in the spleens (Extended Data Fig. 9e–h). Therefore, uPAR CAR T cells persist and expand over the lifespan of the animal, presumably owing to increased antigen stimulation as the frequency of target uPAR positive cells increases over time.

**Extended Data Figure 10 |:**
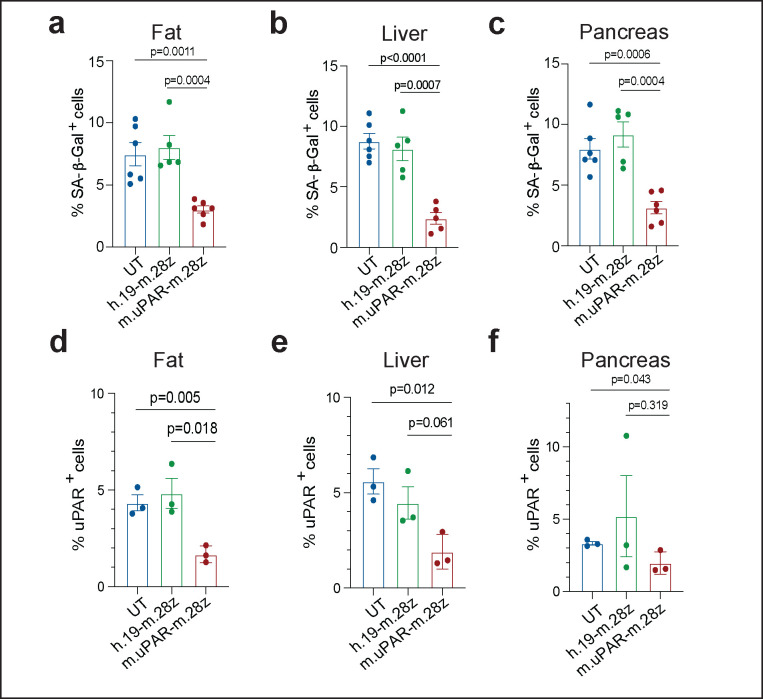
Long-term effect of uPAR CAR T cells on aged tissues. Quantification of SA-β-Gal–positive cells 12 months after cell infusion in **(a)** adipose tissue (n=6 for UT; n=5 for h.19-m.28z; n=6 for m.uPAR-m.28z); **(b)** liver (n=6 for UT; n=5 for h.19-m.28z; n=5 for m.uPAR-m.28z) and **(c)** pancreas (n=6 for UT; n=5 for h.19-m.28z; n=6 for m.uPAR-m.28z). **d-f,** Quantification of uPAR-positive cells in **(d)** adipose tissue, **(e)** liver and **(f)** pancreas 20 days after cell infusion (n=3 per group). Results of 2 independent experiments **(a-c)** and 1 independent experiment **(d-f)**. Data are mean ± s.e.m.; p values from two-tailed unpaired Student’s t-test **(a-f)**.

As was observed in aged animals upon therapeutic treatment, prophylactic uPAR CAR T cell administration in young mice limited metabolic decline in old age. Specifically, uPAR CAR T treated mice had significantly lower fasting glucose levels (Fig. 3c), improved glucose tolerance (Fig. 3d,e) and enhanced pancreatic β cell function as assessed by glucose-stimulated insulin secretion (Fig. 3f) than mice treated with either UT or h.19-m.28z. In terms of fitness, mice that in their youth had been treated with m.uPAR-m.28z CAR T cells, compared with control-treated mice, showed higher exercise capacity at 9 months of age (Fig. 3g,h), although this waned over time (Extended Data Fig. 9i,j). These phenotypes correlated with a significant decrease in both SA-β-Gal-positive and uPAR-positive cells in pancreas, liver, and adipose tissue (Fig. 3i and Extended Data Fig. 10). Taken together, these results show that uPAR CAR T cells can not only treat, but also prevent, features of age-dependent metabolic decline.

### Therapeutic and preventive potential of uPAR CAR T cells in metabolic syndrome

**Extended Data Figure 11 |:**
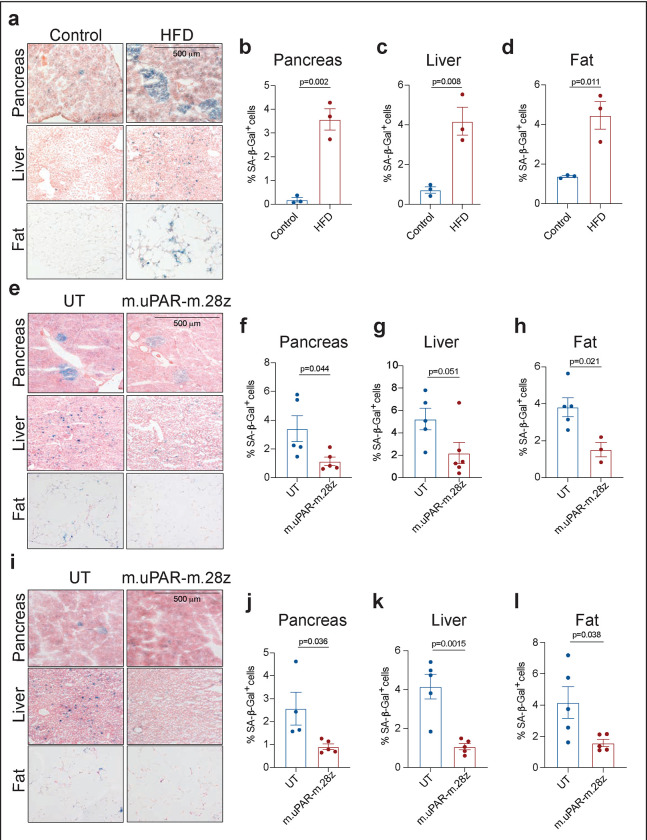
uPAR CAR T cells decrease senescent cell burden in therapeutic and preventive settings in high fat diet. **a,** Representative staining of SA-β-Gal after two months of high fat diet or normal chow diet. **b-d;** Quantification of SA-β-Gal–positive cells in pancreas, liver and adipose tissue after two months of high fat diet or normal chow diet (n=3 for chow; n=3 HFD). **e,** Representative staining of SA-β-Gal 1 month after cell infusion in the experimental scheme depicted in Fig. 4a. **f-h;** Quantification of SA-β-Gal–positive cells in pancreas, liver and adipose tissue 1 month after cell infusion (n=5 for UT; for m.uPAR-m.28z n=5 in pancreas, n=6 in liver and n=3 in adipose tissue). UT, untransduced T cells. **i,** Representative staining of SA-β-Gal 3.5 months after cell infusion in the experimental scheme depicted in Fig. 4j. **j-l,** Quantification of SA-β-Gal–positive cells in pancreas, liver and adipose tissue 3.5 months after cell infusion (UT n=4 in pancreas, n=5 in liver and adipose tissue; for m.uPAR-m.28z n=5). Each panel shows results from 1 experiment. Data are mean ± s.e.m.; p values from two-tailed unpaired Student’s t-test **(b-d; f-h; j-l).**

**Figure 4 |:**
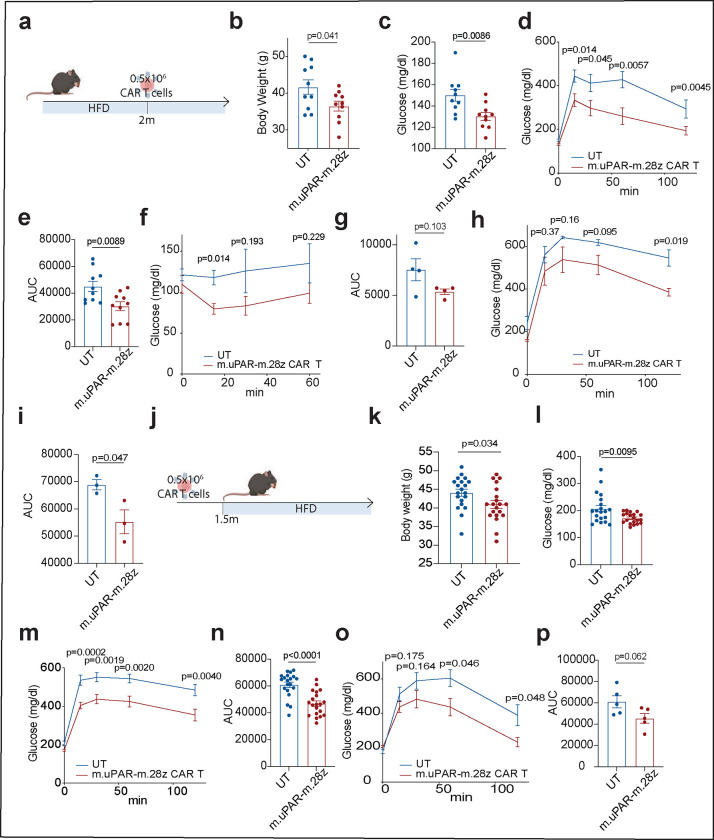
uPAR CAR T cells are therapeutic and preventive in metabolic syndrome. **a,** Experimental scheme for Fig. 4b–i. 3-month-old C57BL/6N mice were treated with high fat diet (HFD) for 2 months followed by intravenous infusion with 0.5×10^6^ m.uPAR-m.28z or untransduced T cells 16h after administration of cyclophosphamide (200 mg/kg). Mice were monitored over time or euthanized 1 month after cell infusion. **b,** body weight 1 month after cell infusion (n=10 mice per group). **c,** Levels of basal glucose after starvation at 1 month after cell infusion (n=10 mice per group). **d,** Levels of glucose before (0 min) and after intraperitoneal administration of glucose (1 g/kg) 1 month after cell infusion (n=10 mice per group). **e,** Area under the curve (AUC) representing the results from **d. f,** Levels of glucose before (0 min) and after intraperitoneal administration of insulin (0.5 units/kg body weight) 1 month after cell infusion (n=4 per group). **g,** AUC representing the results from **f**. Each point represents a single mouse. **h,** Levels of glucose before (0 min) and after intraperitoneal glucose administration (1 g/kg) 2.5 months after cell infusion (n=3 mice per group). **i,** AUC representing the results from **h**. Each point represents a single mouse. **j,** Experimental scheme for Fig. 4k–p. 3-month-old C57BL/6N mice were intravenously infused with 0.5×10^6^ m.uPAR-m.28z or untransduced T cells 16h after administration of cyclophosphamide (200 mg/kg). 1.5 months after infusion, mice were placed on a high fat diet, then monitored over time or euthanized 2 months after the start of the high fat diet. **k,** body weight 3.5 months after cell infusion (n=20 mice per group. **l,** Levels of basal glucose after starvation 3.5 months after cell infusion (n=20 mice per group). **m,** Levels of glucose before (0 min) and after intraperitoneal administration of glucose (1 g/kg) 1 month after cell infusion (n=20 mice per group). **n,** AUC representing the results from **m. o,** Levels of glucose before (0 min) and after intraperitoneal glucose administration (1 g/kg) 5.5 months after cell infusion (n=5 mice per group). **p,** AUC representing the results from **o**. Each point represents a single mouse. **(a-p)**. Results are from 2 independent experiments **(b-e;k-n)** or 1 independent experiment **(f-i; o-p)**. Data are mean ± s.e.m.; p values derived from two-tailed unpaired Student’s t-test **(b-i; k-p)**. Schematics were created with BioRender.com.

Many of the features associated with metabolic syndrome in aged mice can be recapitulated in young animals given a high fat diet^33^ and, indeed, obesity has been described to accelerate the “aging clock”^34^. Indeed, as in aged animals, such treatment leads to the accumulation of senescent cells^7^ (Extended Data Fig. 11a–d). To test the therapeutic potential of uPAR CAR T cells in this context, we modeled metabolic syndrome by feeding mice a high-fat diet (HFD), which induces obesity and metabolic stress^35^. After two months on HFD, mice were treated with 0.5×10^6^ m.uPAR-m.28z CAR T or UT cells and continued on the diet (Fig. 4a). At 20 days after infusion, mice treated with uPAR CAR T cells displayed significantly lower body weight, better fasting blood glucose levels and improvements in both glucose and insulin tolerance compared to controls (Fig. 4b–g). This therapeutic effect persisted through the period of monitoring (2.5m after cell infusion) and was accompanied by decreased senescent cell burden in pancreas, liver and adipose tissue as assessed by SA-β-gal (Fig. 4h,l and Extended Data Fig. 11e–h). Thus, uPAR CAR T therapy produced a similar improvement to metabolic dysfunction in the context of metabolic syndrome in young animals as was observed in naturally aged mice.

**Extended Data Figure 12 |:**
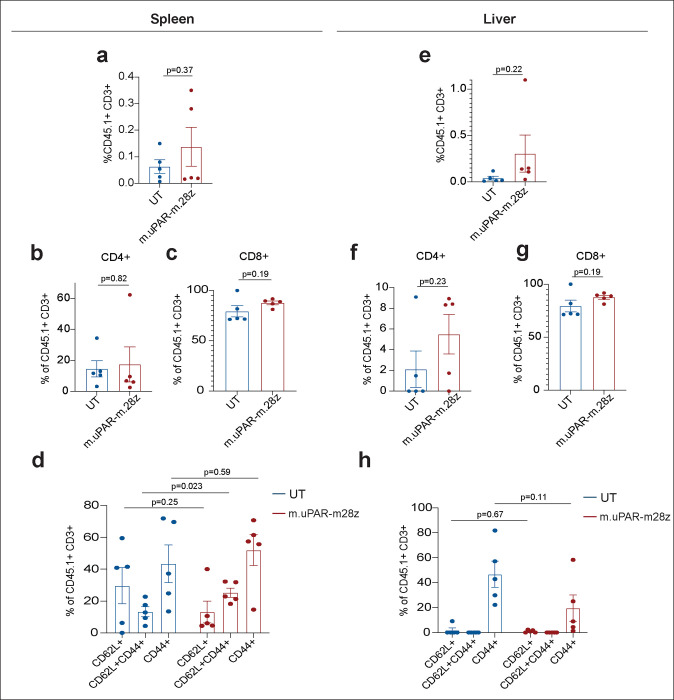
Profile and persistence of uPAR CAR T cells in metabolic syndrome. T cells were assessed in spleen (**a-d**) and liver (**e-h**) 3.5 months after cell infusion in the experimental scheme depicted in Fig. 4j. **a,** Percentage of CD45.1^+^ T cells in the spleen. **b,** Percentage of CD4^+^ cells among CD45.1^+^ T cells in the spleen. **c,** Percentage of CD8^+^ cells among CD45.1^+^ T cells in the spleen. **d,** Percentage of CD45.1^+^ T cells from the spleen expressing differentiation markers CD62L and CD44. **e,** Percentage of CD45.1^+^ T cells in the liver. **f,** Percentage of CD4^+^ cells among CD45.1^+^ T cells in the liver. **g,** Percentage of CD8^+^ cells among CD45.1^+^ T cells in the liver. **h,** Percentage of CD45.1^+^ T cells in the liver expressing differentiation markers CD62L and CD44. Results in each panel are from 1 experiment (n=5 mice per group). Data are mean ± s.e.m.; p values from two-tailed unpaired Student’s t-test.

To test whether prophylactic administration of uPAR CAR T cells could impede the development of metabolic disorders in young mice given HFD, we administered 0.5×10^6^ m.uPAR-m.28z CAR T 1.5 months before placement on HFD (Fig. 4j). Remarkably, m.uPAR-m.28z CAR T cells (but not treatment with UT cells) acted prophylactically to blunt the accumulation of senescent cells over time, an effect that was also associated with decreased weight gain and glucose levels 3.5 months after infusion (Extended Data Fig. 9i–l and Fig. 4k–n). At this time, m.uPAR-m.28z CAR T were detectable and enriched in the spleens and livers of treated mice, they again were composed mostly of CD8 T cells with an effector phenotype (Extended Data Fig. 12). This preventive effect on metabolic dysfunction was sustained for at least 5.5 months after cell infusion despite continuous exposure to high fat diet (Fig. 4o,p).

Overall, these data highlight the contribution of uPAR-positive cells to metabolic dysfunction in aged and obese mice and raise the possibility that targeting these cells through CAR T cells could have therapeutic benefit in humans.

## Discussion

Our study provides proof-of-principle evidence that senolytic cell therapies can ameliorate symptoms associated with physiological aging. We previously showed that uPAR targeting CAR T cells could safely and effectively eliminate senescent cells in the livers of young animals^8^. Here, focusing on metabolic dysfunction as one prominent age-related pathology, we show that: (i) the fraction of uPAR-positive cells increases with age, (ii) that these cells significantly contribute to the senescence burden in aged tissues, (iii) uPAR-positive cells with senescence signatures consist of both immune and non-immune populations, the latter consisting of a range of cell types that are organ dependent, (iv) uPAR CAR T cells can be effective at eliminating uPAR-positive senescent cells; (v) and their effect is not associated with pathology in tissues or alterations of hepatic and renal functional parameters in aged mice. Finally, (vi), the action of uPAR CAR T cells is associated with improved glucose homeostasis and metabolic fitness in both physiological aging and high fat diet. Importantly, at doses used to produce these therapeutic benefits, we noted no overt toxicities of uPAR CAR T cells, which could persist and expand for over 15 months as mice progressed from a youthful to an aged state.

Perhaps the most striking observations of the current work was the ability of uPAR CAR T cells to act prophylactically to blunt age- and diet-induced metabolic decline. Unlike senolytic approaches based on small molecules, uPAR CAR T cells have long-lasting effects after the administration of a single low dose, causing a marked impairment in age- or high fat diet-induced metabolic syndrome when mice were treated during youth or administration of high fat diet, respectively. Our findings are consistent with those of an earlier study that explored vaccination against GPNMB on senescent cells to address age-related pathology^36^, although with our cellular therapy, both effect sizes and duration were substantially larger. In fact, our results demonstrate a protective effect for over a year in the context of physiological aging in the laboratory mouse, a species with an average lifespan of 2 years.

Studies using genetic or pharmacological approaches to senolysis have been equivocal as to whether elimination of senescent cells will significantly extend longevity^3,4,32^. Our current studies are not sufficiently powered to draw conclusions on longevity at this stage. As senescent cells contribute to a range of age-related tissue pathologies, studying the impact of senolysis in aged animals provides an opportunity to interrogate multiple co-morbidities under similar conditions. Future studies will evaluate the potential of uPAR CAR T cells (or other senolytic cell therapies) in additional aging and related tissue-damage pathologies, the latter disease contexts providing a more likely starting point for clinical implementation.

It remains to be determined which of the uPAR-positive cell populations targeted by uPAR CAR T cells are responsible for the improved metabolic function we observe. In other senolytic studies, the elimination of senescent pancreatic beta cells has been linked to improved glucose tolerance^7^. However, there are also reports suggesting that targeting senescent cells in adipose tissue^31^ or even immune-cell senescence^37^ may also play a role. In this regard, recent studies also suggest that the elimination of macrophage populations with senescent features can also improve tissue decline in mice^38,39^. Whether or not these macrophages are truly ‘senescent’ or have an alternative cell state is a topic of debate; regardless, given that we observe a fraction of uPAR-expressing macrophages that also co-express SA-β-gal and senescence-associated transcriptional signatures accumulating in aged tissues it seems likely that their elimination may contribute to the phenotypes we observe.

While the mechanism of action of most current small molecules is often inferred or poorly understood, senolytic CAR T cells have a clear underlying rationale based on the expression of a specific surface antigen. While toxicity issues are invariably a concern, cellular therapy harbors the versatility to simultaneously target several surface antigens through AND gate approaches^16^, modulate persistence through different CAR designs^40^ and/or incorporate safety switches,^41^ all of which provide avenues to mitigate side effects that are not possible through vaccination strategies or small molecule approaches ^41^. Indeed, in another recent report, it has been shown that mice and primates tolerate CAR T cells that target an NK cell ligand that is upregulated on senescent cells and other cell types^42^. Taken together, these efforts could result in the identification of tissue-specific senolytic antigens that could be targeted with cellular therapy to treat different age-related phenotypes. The persistence of the uPAR-targeted CAR T cells and the durability of the effects after a single low-dose treatment highlight the clinical potential of the senolytic CAR T cell approach for the treatment of chronic pathologies.

## Methods

### Mice

All mouse experiments were approved by the MSKCC and/or CSHL Internal Animal Care and Use Committee. All relevant animal use guidelines and ethical regulations were followed. Mice were maintained under specific pathogen-free conditions. The following mice were used: 3- to 4-month-old C57BL/6 mice (purchased from Charles River), 18-month-old C57BL/6 mice (obtained from the National Institute of Aging), and 6-week-old B6.SJL-Ptrca/BoyAiTac (CD45.1 mice) (purchased from Taconic). Mice of both sexes were used at 8–12 weeks of age and 18–20 months of age for the aging experiment, males of 8–12 weeks for the high fat diet experiments and females of 6–10 weeks old for T cell isolation. Mice were kept in group housing. Mice had free access to food and water except during the starvation period before glucose or insulin tolerance testing. Aging mice were fed a normal diet (PicoLab Rodent Diet 20, LabDiet), mice on the high fat diet (HFD) experiments were fed a HFD (TD.06414, 60% of kcal from fat; Envigo). Mice were randomly assigned to the experimental groups.

### Flow cytometry

For in vivo sample preparation, livers were dissociated using the MACS liver dissociation kit (Miltenyi Biotec, 130–1-5–807), filtered through a 100-μm strainer and washed with PBS, and red blood cells were lysed by an ACK (ammonium–chloride–potassium) lysing buffer (Lonza). Cells were washed with PBS, resuspended in FACS buffer and either used for immediate analysis or fixed with Fixation Buffer (BD Biosciences; 554655) according to the manufacturer’s instructions and used for later analysis. Spleens were mechanically disrupted with the back of a 5-ml syringe, filtered through a 40-μm strainer and washed with PBS and 2 mM EDTA, then red blood cells were lysed by ACK lysing buffer (Lonza). Gonadal adipose tissue was dissociated as described^43^. In short, adipose tissue was isolated and placed in a digestion solution consisting of 4 mg/ml collagenase, type II (Sigma) in DPBS (Life Technologies) supplemented with 0.5% BSA (Sigma) and 10 mM CaCl_2_ digested at 37° C for 20 min in a rotational shaker (200 rpm). Afterwards, samples were mechanically dissociated with a 10-ml serological pipette, filtered through a 40-μm strainer and washed with PBS and 2 mM EDTA, then red blood cells were lysed by ACK lysing buffer (Lonza). Pancreata were placed into cold DMEM with 10% FBS and penicillin and streptomycin. The pancreata were minced in this media on ice into 2- to 4-mm fragments so that they would pass through the end of 1-ml pipette tip with ease. The minced tissue was collected in a 15-ml Falcon tube and dissociated in 100 mg/ml Dispase (Life Tech., cat. 17105041), 20 mg/ml collagenase P (Roche, cat. 11249002001) and 1 mM EDTA for 20 minutes on a heated rocker at 37° C (Eppendorf). After 20 minutes, 5 ml of DMEM with 10% FBS was added to quench the reaction. The supernatant was removed and filtered through a 100-μm filter( VWR). Next, 5 ml of dissociation media consisting of 100 mg/ml Dispase (Life Tech., cat. 17105041), 20 mg/ml collagenase P (Roche, cat. 11249002001) and 1 mM EDTA was added prior to replacing the 15-ml tube into the heated rocker for 20 minutes. The reaction was quenched again after 20 minutes with media and filtered via a 100-μm filter. The dissociated cells were spun at 500 rcf for 10 minutes in a swinging bucket centrifuge. The supernatant was discarded and the cells were resuspended in ACK lysis buffer for 2–4 minutes in ice. Cells were washed with PBS, resuspended in FACS buffer and either used for immediate analysis or fixed with Fixation Buffer (BD Biosciences;554655) and used for later analysis.

Fc receptors were blocked using FcR blocking reagent, mouse (Miltenyi Biotec). The following fluorophore-conjugated antibodies were used in the indicated dilutions: Myc-tag AF647 (clone 9B11, Cell Signaling Technology, 2233S, lot 25, 1:50), m.CD45.1 BV785 (clone A20, Biolegend, 110743, lot B347719, 1:100), m.CD45.2 BV785 (clone 104, Biolegend, 109839, lot B343292, 1:100), mCD3 AF488 (clone 17A2, Biolegend, 100210, lot B284975, 1:100), mCD4 BUV395 (clone GK1.5, BD, 563790, lot 1097734, 1:50), mCD8 PE-Cy7 (clone 53–6.7, Biolegend, 100722, lot B312604, 1:50), mCD62L BV421 (clone MEL-14, Biolegend, 104435, lot B283191, 1:50), mCD44 APC Cy7 (clone IM7, BD Pharminogen, 560568, lot 1083068, 1:100), mCD3 BV650 (clone 17A2, Biolegend, 100229, lot B350667, 1:100),mCD19 BV650 (clone1D3, BD Biosciences, 563235, lot 1354015 1:100), mNKp46 BV650 (clone 29A1.4, Biolegend, 137635, lot B298809 1:100), mCD11b BUV395 (clone M1/70, BD Biosciences, 563553, lot 0030272 1:50), mLy-6C APC-Cy7 (clone HK1.4, Biolegend, 128026, lot B375238 1:100), mly6G BV605 (clone 1A8, BD Biosciences, 563005, lot2144780 1:100), m.uPAR AF700 (R&D systems, FAB531N, lot 1656339, 1:50), m.uPAR PE (R&D systems, FAB531P, lot ABLH0722051, 1:50), mF4/80 PE-eFluor610 (Clone BM8, Invitrogen, 61–4801-82, lot 2338698, 1:100).7-AAD (BD, 559925, lot 9031655, 1:40) or Ghost UV 450 Viability Dye (13–0868-T100, Tonbo Biosciences lot D0868083018133, 1ul/ml) was used as viability dye. Flow cytometry was performed on a LSRFortessa instrument (BD Biosciences), FACS was performed on a SONY SH800S cell sorter and data were analyzed using FlowJo (TreeStar).

### Single cell RNA-seq:

Sequencing data was demultiplexed, mapped, and processed into gene/cell expression matrices using 10X Genomics’ Cell Ranger software v7.1.0 (https://support.10xgenomics.com/single-cell-gene-expression/software/pipelines/latest/what-is-cell-ranger). Gene expression reads were aligned to the mouse reference genome version gex-mm10–2020-A, available from the 10X Genomics website. We kept cells using “min.cells > 10, nFeature_RNA > 500, nCount_RNA > 2500, percent.mt < 15”. Gene expression count data were normalized using SCTransform to regressed out percent mitochondrial RNA. The R package BBKNN was used to remove batch effects between mouse samples, and 0.5 was used as expression cutoff to define uPAR High cell populations. Clusters were identified using resolution = 0.8, and cell types were annotated using R packages celldex, SingleR, Azimuth, and custom gene sets^24,25^. Known markers for each cell type were plotted using DotPlot function in Seurat. Senescence gene sets from^21,28^ were used to calculate signature scores using AddModuleScore function in Seurat, and a signature score cutoff of 0.05 was used to define Senescence High cell populations.

### Isolation, expansion and transduction of mouse T cells

B6.SJL-Ptrca/BoyAiTac mice (CD45.1 mice) were euthanized and spleens were collected. After tissue dissection and red blood cell lysis, primary mouse T cells were purified using the mouse Pan T cell Isolation Kit (Miltenyi Biotec). Purified T cells were cultured in RPMI-1640 (Invitrogen) supplemented with 10% FBS (HyClone), 10 mM HEPES (Invitrogen), 2 mM l-glutamine (Invitrogen), MEM non-essential amino acids 1× (Invitrogen), 55 μM β-mercaptoethanol, 1 mM sodium pyruvate (Invitrogen), 100 IU ml^−1^ recombinant human IL-2 (Proleukin; Novartis) and mouse anti-CD3/28 Dynabeads (Gibco) at a bead:cell ratio of 1:2. T cells were spinoculated with retroviral supernatant collected from Phoenix-ECO cells 24 h after initial T cell activation as described^44,45^ and used for functional analysis 3–4 days later.

### Genetic modification of T cells

The mouse SFG γ-retroviral m.uPAR-m28z plasmid has been described^8^. The mouse SFG γ-retroviral h.19-m28z plasmid^8^ was constructed by stepwise Gibson assembly (New England BioLabs) using the amino acid sequence for the single-chain variable fragment (scFv) specific for human CD19 of the SFG-1928z backbone^46^ and cloned into the backbone of the SFG γ-retroviral m.uPAR-m28z plasmid^8^. In both constructs the anti-mouse uPAR scFv or anti-human CD19 scFv is preceded by a mouse CD8A leader peptide and followed by the Myc-tag sequence (EQKLISEEDL), mouse CD28 transmembrane and intracellular domain and mouse CD3z intracellular domain^44,45^. Plasmids encoding the SFGγ retroviral vectors were used to transfect gpg29 fibroblasts (H29) to generate VSV-G pseudotyped retroviral supernatants, which were used to construct stable retrovirus-producing cell lines as described^44,46^.

### Glucose tolerance testing

Blood samples from mice fasted 8–12h were collected at 0, 15, 30, 60 and 120 minutes after intraperitoneal injections of glucose (Sigma Aldrich) (2 g/kg body weight). Insulin was measured from serum collected at the 0- and 15-minute time points. Concentrations were determined using the UltraSensitive Mouse Insulin ELISA kit (Crystal Chem, 90080).

### Insulin tolerance testing

Blood samples from mice fasted 4h were collected at 0,15, 30 and 60 minutes after intraperitoneal injections of insulin (Humulin R; Eli Lilly) (0.5 units/kg body weight).

### Histological analysis

Tissues were fixed overnight in 10% formalin, embedded in paraffin and cut into 5-μm sections. Sections were subjected to hematoxylin and eosin (H&E) staining. Immunohistochemical staining was performed following standard protocols. The following antibodies were used: anti-mouse uPAR (R&D, AF534, lot DCL0521042, 1:50), Horse anti-goat IgG (Vector laboratories, 30116; lot ZH0526). Three fields per section were counted per sample with ImageJ and averaged to quantify the percentage of uPAR+ area per field. SA-β-gal staining was performed as previously described^47^ at pH 5.5 for mouse tissues. Specifically, fresh frozen tissue sections were fixed with 0.5% glutaraldehyde in phosphate-buffered saline (PBS) for 15 min, washed with PBS supplemented with 1 mM MgCl_2_ and stained for 5–8 h in PBS containing 1 mM MgCl_2_, 1 mg ml^−1^ X-gal, 5 mM potassium ferricyanide and 5 mM potassium ferrocyanide. Tissue sections were counterstained with eosin. Three fields per section were counted with ImageJ and averaged to quantify the percentage of SA-β-gal+ area per field.

### Immunofluorescence analysis

For the fluorescent SA-β-gal labelling, tissue slides were exposed to the C12RG substrate at 37°C according to manufacturer’s instructions (ImaGene Red C12RG lacZ Gene Expression Kit, Molecular Probes, I2906)^48,49^. Subsequently, for IF analysis, slides were fixed with 4% PFA for 10 minutes at room temperature and proceed with regular IF as performed following standard protocols and previously described^8^. The following antibodies were used: anti-mouse uPAR (R&D, AF534, 1:100) and anti-mouse F4/80 (Bio Rad, CI:A3–1). For quantification 5 high power fields per section were counted and averaged to quantify the percentage of SA-β-gal+, uPAR+ and F4/80+ per DAPI positive cells. For co-localization analysis Pearson coefficient was calculated using ImageJ.

### Exercise capacity testing

Exercise capacity was assessed using a motorized treadmill (model 1050 EXER 3/6; Columbus Instruments, Columbus, OH). For 3 days prior to testing, mice were acclimatized to the treadmill (the mice walked on the treadmill at 10 m/min for 10 to 15 minutes per day). Following acclimatization, all mice underwent exercise capacity tests on consecutive days. Tests began with mice walking at 10 meters/min with speed increased by 2 meters/min every two minutes until exhaustion (mice could no longer achieve treadmill running speed despite repeated encouragement). The primary end points were time to exhaustion and maximum speed.

### Blood measurements

Complete blood counts with differentials were performed using an automated hematology analyzer (IDEXX Procyte DX, Columbia, Missouri). For serum chemistry, blood was collected in tubes containing a serum separator. The tubes were then centrifuged, and the serum was obtained for analysis. Serum chemistry was performed by the LCP on a Beckman Coulter AU680 analyzer (Beckman Coulter Life Sciences, Brea, CA). For cytokine analysis, plasma was collected and samples were processed and measured by Eve Technologies.

### Pathology

Mice submitted for postmortem examination were euthanized by CO_2_ asphyxiation and cardiac exsanguination. Complete necropsies were performed at the Laboratory of Comparative Pathology (MSK, the Rockefeller University, and Weill Cornell Medicine). Representative sections were taken from all organ systems including heart, thymus, lungs, esophagus, trachea, thyroid glands, spleen, pancreas, liver, gallbladder, kidneys, adrenal glands, stomach, duodenum, jejunum, ileum, cecum, colon, lymph nodes (mesenteric and submandibular), salivary glands, skin (trunk and head), urinary bladder, epididymides, testes, prostate, seminal vesicles, uterus, cervix, vagina, ovaries, oviducts, spinal cord, vertebrae, sternum, femur, tibia, stifle joint, skeletal muscle, nerves, skull, nasal cavity, oral cavity, teeth, ears, eyes, pituitary gland, and brain. Sections were fixed in 10% neutral-buffered formalin, processed in alcohol and xylene, embedded in paraffin, sectioned (5 μm thick) and stained with hematoxylin and eosin. The skull, spinal column, sternum, and hindlimb were decalcified in a formic acid and formaldehyde solution (Surgipath Decalcifier I, Leica Biosystems, Wetzlar, Germany) before processing. H&E-stained tissue sections were evaluated by a board-certified veterinary pathologist (S.E.C.). Representative images were captured using a brightfield BX45 microscope with a DP26 camera and cellSens (version 1.18) Dimension software (Olympus America, Center Valley, Pennsylvania).

### Statistical analysis and figure preparation

Data are presented as mean ± s.e.m. Statistical analysis was performed by Student’s t-test or Mann Whitney test using GraphPad Prism v.6.0 or 7.0 (GraphPad software). No statistical methods were used to predetermine sample size in the mouse studies, and mice were allocated at random to treatment groups. Figures were prepared using BioRender.com for scientific illustrations and Illustrator CC 2019 (Adobe).

## Extended Data

**Extended Data Figure 13 |:**
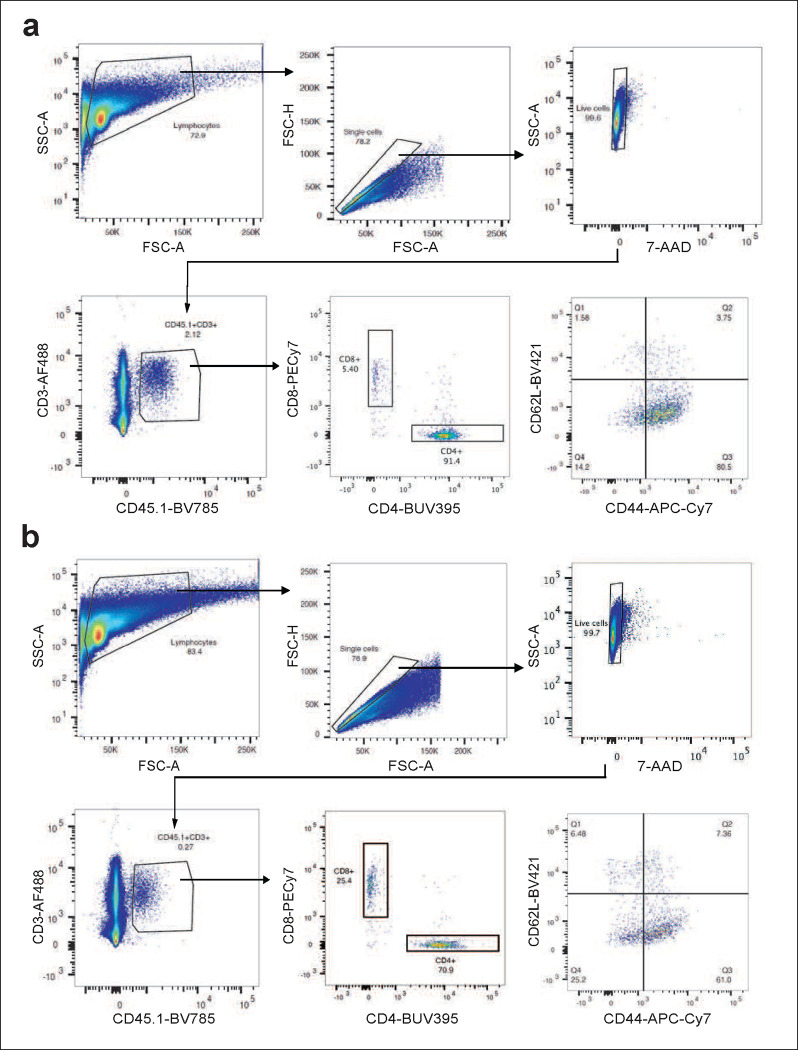
Gating strategies. **a,b,** Representative flow cytometry staining of m.uPAR-m.28z **(a)** or untransduced T cells **(b)** obtained from the spleens of mice 20 days after cell infusion as depicted in Fig. 2l. Shown are results of 1 independent experiment (n=3 mice for untransduced T cells; n=4 mice for m.uPAR-m.28z).
